# The importance of optical optimization in whole slide imaging (WSI) and digital pathology imaging

**DOI:** 10.1186/1746-1596-3-S1-S1

**Published:** 2008-07-15

**Authors:** Yukako Yagi, John R Gilbertson

**Affiliations:** 1 Department of Pathology, Harvard Medical School, Boston, MA, USA

## Abstract

In the last 10 years, whole slide imaging (WSI) has seen impressive progress not only in image quality and scanning speed but also in the variety of systems available to pathologists. However, we have noticed that most systems have relatively simple optics axes and rely on software to optimize image quality and colour balance. While much can be done in software, this study examines the importance of optics, in particular optical filters, in WSI.

Optical resolution is a function of the wavelength of light used and the numerical aperture of the lens system (Resolution = (f) wavelength/2 NA). When illumining light is not conditioned correctly with filters, there is a tendency for the wavelength to shift to longer values (more red) because of the characteristics of the lamps in common use. Most microscopes (but remarkably few WSI devices) correct for this with ND filter for brightness and Blue filter (depends on the light source) for colour correction.

Using H&E slides research microscopes (Axiophot, Carl Zeiss MicroImaging, Inc. NY. Eclipse 50i., Nikon Inc. NY) at 20×, an attached digital camera (SPOT RT741 Slider Color, Diagnosis Instruments., MI USA), and a filter set, we examined the effect of filters and software enhancement on digital image quality. The focus value (as evaluated by focus evaluation software developed in house and SPOT imaging Software v4.6) was used as a proxy for image quality. Resolution of tissue features was best with the use of both the Blue and ND filters (in addition to software enhancement). Images without filters but with software enhancement while superficially good, lacked some details of specimen morphology and were unclear compared with the images with filters.

The results indicate that the appropriate use of optical filters could measurably improve the appearance and resolution of WSI images.

## Background

Whole slide imaging (WSI) has made impressive progress over the past ten years not only in technology (faster scanning and better image quality) but also as the number of applications in Pathology (such as image analysis and enhancement). Further, developers are also looking at WSI as a front end for computer aided diagnosis tools and anatomic pathology laboratory information systems in conjunction of other new relatively new technologies. In this circumstance, the quality and accuracy of image data (how accurate) is becoming increasingly important.

The technologies in WSI scanners include optics, electronics, mechanical systems (such as automated slide loaders and stages), as well as computer software such as image enhancement and compression. Every component of every technology is important and must work well with all others. In many systems today, optics is relatively simple and images are 'enhanced' using software approaches such as white balance correction.

In this paper, we focus on the optimization of optics, especially optical colour balance and the effect of colour balance effects on focus and image quality.

## Methods

When the microscope is used for diagnosis, there is the tendency to turn the light down without using a filter because the light is too bright. However, turning down the voltage affects the resolution and colour of the microscope image.

Optical resolution is a function of the wavelength of light used and the numerical aperture of the lens system (Resolution = (f) wavelength/2 NA). When illumining light is not conditioned correctly with filters, there is a tendency for the wavelength tends to shift to larger values (more red) because of the characteristics of the lamps in common usage. Most microscopes (but remarkably few WSI devices) correct for this with ND filter for brightness and Blue filter (depends on the light source) for colour correction.

To study the importance of optical optimization in WSI and digital pathology imaging, we used two microscope imaging systems and one WSI scanner that could be fitted with filters temporarily for this experiment (most WSI scanners do not accept any hardware modification at user end).

### Microscope imaging system

Using H&E slides and two research microscopes (Axiophot, Carl Zeiss MicroImaging, Inc. NY, Eclipse 50i., Nikon Inc. NY) at 20×, an attached digital camera (SPOT RT741 Slider Colour, Diagnosis Instruments., MI USA), and a filter set, we examined the effect of filters and software enhancement (such as white balance, colour correction, or gamma correction) (SPOT imaging Software v4.6) on digital image quality. The focus value (as evaluated by focus evaluation software developed in house and SPOT imaging Software v4.6) was used as a proxy for image quality. The image acquisition conditions examined in this paper are in Table 1.

**Table 1:** The combination of image acquisition. The combination of image acquisition conditions for each experiment: x = used, o = not used.

	Blue Filter	ND Filter	Camera colour correction	Software colour correction
1	x	x	x	x
2	x	o	x	x
3	o	x	x	x
4	x	x	o	x
5	x	x	x	o
6	o	o	x	x
7	o	o	o	x
8	o	o	x	o
9	o	o	o	o

### WSI scanner

Film type filters were inserted under the glass slide during scanning. The sky blue filter was selected with the consideration of the scanner's right source. We examined the difference between the two with the filter and software white balance and without filter (default setting of the scanner).

## Results

### Microscope imaging system

With both microscope systems, resolution of tissue features was best with the use of both the Blue and right ND filters in addition to software enhancement.

The experimental conditions that gave the best results, in order of quality were 9 > 8, 7 > 6 > 5, 4 > 3 > 2 > 1 (See Table 1 for description of conditions)

**Figure 1:**
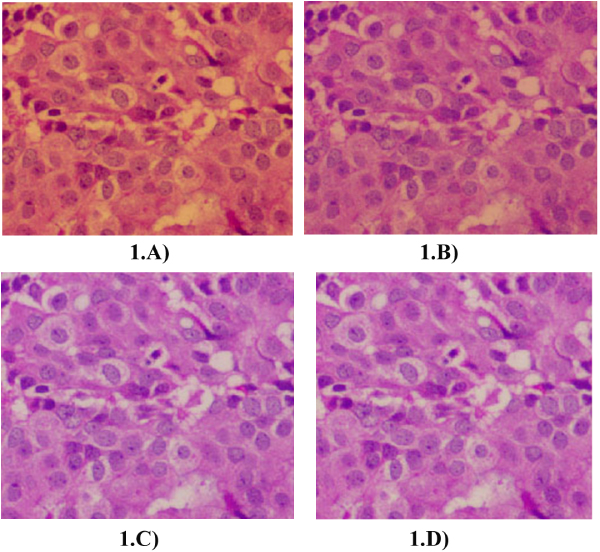
**The importance of optical filters**. A) Experimental condition 3 (Table 1), with NB filter only. Without Blue but with ND filters: F#: 56. B) Experimental condition 6 (Table 1), no filters. Without either Blue or ND filters F#: 50. C) Experimental condition 2 (Table 1), Blue filter only. With Blue, without ND filter F#:60. D) Experimental condition 1 (Table 1), both ND and Blue filters. Software enhancement, camera correction are the same in all four images. Higher value of F# indicates a higher effective resolution. With Blue, without ND filter F#:60.

Figure 1 shows some of results. The "F#" is the numerical output of the image quality evaluation software (in this case, the SPOT imaging Software v4.6). A higher F# indicates a better effective resolution. The F# indicates a better result with the use of optical filters, a result backed up by visual examination of the images. Software and camera settings were the same in all cases. The use of filters seems to increase image quality with or without the use of software enhancements like white balance.

### WSI scanner

**Figure 2:**
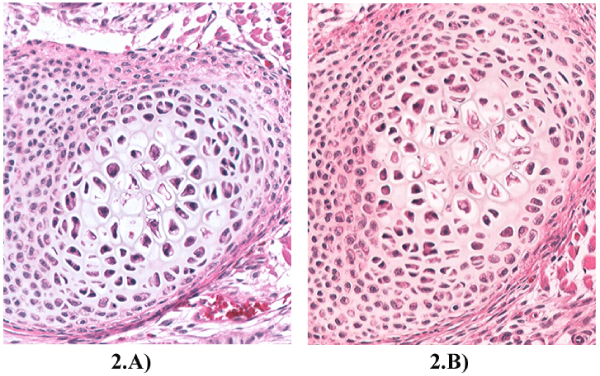
**Results of adding a sky-blue filter to a WSI device**. A) With Sky-blue filter and white balance, B) WSI Scanner without filters (default).

Figure 2 shows the results of applying a filter to a WSI scanner. A. is a scan with the sky-blue filter while B is a scan without a filter (the default scanner setting). A. shows the colour of the real glass slide.

## Conclusion

The results indicate that the appropriate use of optical filters could measurably improve the resolution and quality of WSI images. The use of software enhancement only (without the use of filters) resulted in images that while superficially had a good appearance, were missing some of details of the specimen and were unclear compared with the images when the filters were added.

